# Identification of key gene networks related to the freezing resistance of apricot kernel pistils by integrating hormone phenotypes and transcriptome profiles

**DOI:** 10.1186/s12870-022-03910-4

**Published:** 2022-11-15

**Authors:** Xiaojuan Liu, Huihui Xu, Dan Yu, Quanxin Bi, Haiyan Yu, Libing Wang

**Affiliations:** State Key Laboratory of Tree Genetics and Breeding, Research Institute of Forestry, Chinese Academy of Forestry, Beijing, 100091 China

**Keywords:** Apricot kernel, Freezing stress, Hormone, Transcriptome, Auxin, Gene network

## Abstract

**Background:**

Apricot kernel, a woody oil tree species, is known for the high oil content of its almond that can be used as an ideal feedstock for biodiesel production. However, apricot kernel is vulnerable to spring frost, resulting in reduced or even no yield. There are no effective countermeasures in production, and the molecular mechanisms underlying freezing resistance are not well understood.

**Results:**

We used transcriptome and hormone profiles to investigate differentially responsive hormones and their associated co-expression patterns of gene networks in the pistils of two apricot kernel cultivars with different cold resistances under freezing stress. The levels of auxin (IAA and ICA), cytokinin (IP and tZ), salicylic acid (SA) and jasmonic acid (JA and ILE-JA) were regulated differently, especially IAA between two cultivars, and external application of an IAA inhibitor and SA increased the spring frost resistance of the pistils of apricot kernels. We identified one gene network containing 65 hub genes highly correlated with IAA. Among these genes, three genes in auxin signaling pathway and three genes in brassinosteroid biosynthesis were identified. Moreover, some hub genes in this network showed a strong correlation such as protein kinases (PKs)-hormone related genes (HRGs), HRGs-HRGs and PKs-Ca^2+^ related genes.

**Conclusions:**

Ca^2+^, brassinosteroid and some regulators (such as PKs) may be involved in an auxin-mediated freezing response of apricot kernels. These findings add to our knowledge of the freezing response of apricot kernels and may provide new ideas for frost prevention measures and high cold–resistant apricot breeding.

**Supplementary Information:**

The online version contains supplementary material available at 10.1186/s12870-022-03910-4.

## Background

Apricot kernel, belonging to the Rosaceae family, is a general designation of apricot varieties producing almond. Apricot kernels mainly conclude large flat apricots (*Prunus armeniaca* × *Prunus sibirica*) that produce sweet almonds and Siberian apricots (*Prunus sibirica*) that produce bitter almonds. Apricot kernel is primarily distributed in northern China and is an important economic forest species and woody grain and oil resource, with an oil content of up to 54% [1]. Because of a low freezing point (− 20 °C) almond oil can be used as a raw material for producing industrial oils such as premium lubricants and biodiesel [2]. However, there is still the problem of unstable yield from apricot kernels. Because of the early blooming of apricot, its flower organs and even young fruits are vulnerable to late frost damage in spring, resulting in reduced production or even no harvest [3]. Therefore, spring frost has become the main factor restricting the production and development of apricot kernel.

Spring frost occurs when the temperature drops below 0 °C after warming up in spring, thus seriously endangering the yield of agricultural and forestry plants, such as maize, wheat, apple and apricot [4–7]. Moreover, with the trend of global warming, the temperature increases in early spring, which makes plants more susceptible to spring frost. Spring frost has caused considerable economic losses to China [7, 8]. Some prevention measures used to resist spring frost have been taken, but the effects are not substantial. New defense measures and cold-resistant varieties are of considerable importance for economic plants to reduce economic and production losses.

When a plant encounters spring frost, a series of sophisticated cold acclimation mechanisms can be activated that allow the plant to adapt to freezing stress [9]. The ICE1-CBF-COR transcriptional cascade is critical for cold acclimation in higher plants [10, 11]. Meanwhile, many messenger molecules (e.g., Ca^2+^ and ROS), protein kinases (SnRKs and RLKs) and transcription factors (TFs) (NAC and bHLH) have been confirmed to regulate cold stress signaling at transcriptional, posttranscriptional and posttranslational levels [11–13]. For example, protein kinase OST1/SnRK2.6, which was activated by cold stress positively regulated freezing tolerance via phosphorylating ICE1, BTF3s and PUB25/26 to enhance *CBF* expression [14]. Recently, CabHLH79 TF acting upstream of CaNAC035 positively regulated cold tolerance via regulation of ROS-related and cold responsive genes (e.g., *CBF1A*) in pepper [15]. In addition, growing evidence shows that plant hormones, as small endogenous signaling molecules, play important roles in regulating plant freezing tolerance. Cold temperatures can impact some hormone content in plants by regulating the expression of biosynthetic genes, such as auxin and brassinosteroid (BR) [16]. Meanwhile, BR and auxin, via their key components (e.g., BIN2 and AUX/IAA) of signaling pathways, can modulate plant development under cold stress [16]. A previous study found that auxin is directly integrated into the DREB/CBF stress pathway via regulation of *Aux/IAA* genes [17]. The mutant of *SLR/IAA14*, a transcriptional repressor of auxin signaling, increased the sensitivity to cold stress via microRNA169 [18]. In addition, hormone-hormone crosstalk also have important roles in the response to cold stress, such as JA-abscisic acid (ABA) crosstalk and JA-gibberellin (GA) crosstalk [19, 20].

In apricot, the external application of plant hormones, such as ABA and SA, can improve the spring frost resistance of the floral organ [21, 22]. However, the function and interaction of hormones on the freezing resistance of apricot kernel and its underlying mechanism is not very clear. In this study, to analyze the hormone-mediated response mechanism of apricot kernel’s floral organ during spring frost stress, the pistils of two apricot kernel cultivars (cold-tolerant ‘Weixuan 1’ (CtW) and cold-sensitive ‘Longwangmao’ (CsL)) were used for transcriptome and hormone analyses. The endogenous hormones that differed between CtW and CsL and their highly correlated differentially expressed genes (DEGs) were analysed. This study aims to provide new insights into the mechanisms in response to freezing stress and a direction for preventing spring frost in apricot.

## Materials and methods

### Plant materials and cold treatment

The two main apricot kernel cultivars (CtW and CsL) that belonging to large flat apricots (*Prunus armeniaca* × *Prunus sibirica*) were used to evaluate spring frost resistance. CsL was recognized by the National Crop Variety Examination and Approval Committee in 1993, and the variety registration number is GS14010 − 1992. CtW was approved by the Forest Variety Examination and Approval Committee of the State Forestry Administration, and the improved variety number is S-SV-PA-018-2010. The spring frost resistance of CtW during the flowering stage is stronger than CsL [23–25]. CtW was selected from CsL via bud mutation [23], which reduced the difference in their genetic background. The flower branches of CtW and CsL were collected at the Apricot Germplasm Resource in Shanxi, China, at the end of December when the flower buds were in hibernation. The flower branches were hydroponically cultured in an incubator (20 °C) until the flowers were in full bloom; then, the whole flower branches were placed in a low-temperature incubator for low-temperature treatment. Low-temperature treatment was set at − 2 °C, − 3 °C and − 4 °C based on the half lethal temperature of CtW and CsL [25], and the method of low temperature treatment was to reduce the temperature from 20 °C to 2 °C at a rate of 10 °C/0.5 h and then reduce to the treatment temperature at a rate of 3 °C/h, which aimed to simulate natural cooling, and maintain it at the treatment temperature for 1 h [26]. The low temperature treated and untreated pistils were collected in liquid nitrogen and stored at − 80 °C prior to the hormone contents analysis, transcriptome sequencing and quantitative real-time PCR (qRT-PCR) analysis.

The unopened flowering branches of CsL were treated with various hormone solutions (6.8 mmol/L IAA inhibitor (PP333) + 2.17 mmol/L SA, 2.17 mmol/L SA, 6.8 mmol/L PP333, 15 mg/L tZ and 100 mg/L JA) for 2 weeks before the arrival of spring frost, every 3 − 4 days. Different hormone treatments were performed on different flowering branches of one tree, and five trees were selected for these treatments as biological replicates.

### Hormone content analysis

Samples from the pistils of CsL (CsL1 (20 °C), CsL2 (− 2 °C), CsL3 (− 3 °C) and CsL4 (− 4 °C)) and CtW (CtW1 (20 °C), CtW2 (− 2 °C), CtW3 (− 3 °C) and CtW4 (− 4 °C)) containing three biological replicates were used for hormone content detection. The powder samples were extracted in methanol/water/formic acid (15:4:1, V/V/V), and then the extracts were redissolved in 80% methanol (V/V) solution after concentration. The extracts were analyzed on an LC (Shim-pack UFLC SHIMADZU CBM30A, http://www.shimadzu.com.cn/)- MS/MS (Applied Biosystems 6500 Quadrupole Trap, http://www.appliedbiosystems.com.cn/) system (the temperature of electrospray ionization was 500 °C, mass spectrum voltage 4500 V, curtain gas (CUR) 35 psi, the collision-activated dissociation parameter was set to medium). The qualitative and quantitative analysis of hormones were obtained by Analyst 1.6.1 software based on different concentrations of hormone standards by Wuhan Metwell Biotechnology Co., Ltd. (Wuhan, China).

### RNA exaction and sequencing

Total RNA from 24 samples (CsL1, CsL2, CsL3, CsL4, CtW1, CtW2, CtW3 and CtW4) containing three biological replicates (every five flower branches were as a biological repeat, which contain 50–70 pistils) was extracted. The purity and integrity of RNA were analyzed via agarose gel electrophoresis, a Nano Photometer spectrophotometer (Implen, CA, USA) and an Agilent 2100 Bioanalyzer (Agilent Technologies, CA, USA), and the RNA concentration was detected using a Qubit 2.0 Fluorometer (Life Technologies, CA, USA). The cDNA libraries were constructed from RNA samples and paired-end sequencing was performed on an Illumina HiSeq platform (Illumina, San Diego CA, USA) at Wuhan Metwell Biotechnology Co., Ltd. (Wuhan, China). After raw data filtering (the reads with the adapter sequences, the reads containing more than 10% ambiguous “N” bases and 50% low-quality bases (Q < =5) were removed), sequencing error rate inspection and GC content distribution inspection, the clean reads were obtained. The HISAT2 software was used to align the clean reads with the reference genome sequence [27] (https://www.rosaceae.org/species/prunus_armeniaca/genome_v1.0) to obtain the position and sequence feature information.

### Identification of DEGs and enrichment analyses

DEG analysis between two groups was conducted by the DESeq2 R package. The counts data were pre-filtered with parameter 2 to remove the lines with very little expression. The resulting *P* values were adjusted by Benjamini–Hochberg’s method to obtain the false discovery rate (FDR). Genes with |log2Fold Change| > =1 and FDR < 0.01 were considered DEGs. The GO and KEGG functional annotation files of reference genome was downloaded from https://www.rosaceae.org/species/prunus_armeniaca/genome_v1.0, which were analyzed using InterProScan and KAAS (KEGG Automatic Annotation Server: http://www.genome.jp/kegg/kaas/), respectively [28]. The KEGG enrichment analyses were performed by the Omicshare online program (https://www.omicshare.com/tools/) and the pathways with *p*-values ≤0.05 were considered to be significantly enriched.

### Weighted gene coexpression network and hub gene analyses

Weighted gene coexpression network analysis (WGCNA) was performed using the WGCNA package with default parameters in R according to a previous method [29]. The phenotype of hormone content and FPKM value of DEGs between CtW and CsL (CsL2 vsCtW2, CsL3 vs CtW3 and CsL4 vs CtW4) were used for WGCNA. The FPKM values were log-transformed using *log2(x + 1)*, and then an adjacency matrix was constructed by calculating Pearson’s correlations between each pair of genes. A soft thresholding power of β = 18 was chosen to ensure a scale-free network using the gradient method with the scale free topology model fit (*R*^*2*^) of 0.90. The adjacency matrix was converted into a topological overlap matrix (TOM) using WGCNA package. Co-expression genes modules were identified as clusters from the dendrogram based on TOM using the cutreeDynamic function with a minimum module size of 30 genes. DEGs with similar expression patterns were grouped into one module. The module eigengenes (MEs) were computed for each module and the first principal gene in the module was defined as the ME representing the gene expression profile within a module. An ME distance threshold of 0.20 was used to further merge highly correlated modules. Based on the identified modules, the correlations between MEs and hormone content were estimated. The modules (the absolute values of correlation greater than 0.80 and a significance level less than 0.01) were considered highly related to corresponding hormone. After module confirmation, DEGs with kME (intramodule connectivity) > 0.9 and gene significance (GS) > 0.2 were defined as hub genes. Visualization of hub gene co-expression networks was performed with Cytoscape software (v3.7.2).

### qRT-PCR validation of DEGs

Total RNA was extracted from the above treated samples using RNA Plant Plus Reagent (Tiangen, Beijing, China), and cDNA was synthesized with a PrimeScript first-strand cDNA synthesis kit (Takara, Dalian, China). The genes were randomly selected from DEGs in CtW and CsL. qRT-PCR was performed with KAPA SYBR FAST qPCR Master Mix (Kapa Biosystems, USA) as described in the instructions. The expression levels of genes were normalized to *18S* and calculated using the 2^−ΔΔCt^ method. Three biological replicates were used for qRT-PCR analysis. The primers used in qRT-PCR are listed in Table S1.

## Results

### Hormone responses in the pistils of freezing stress–treated apricot kernels

**Fig. 1:**
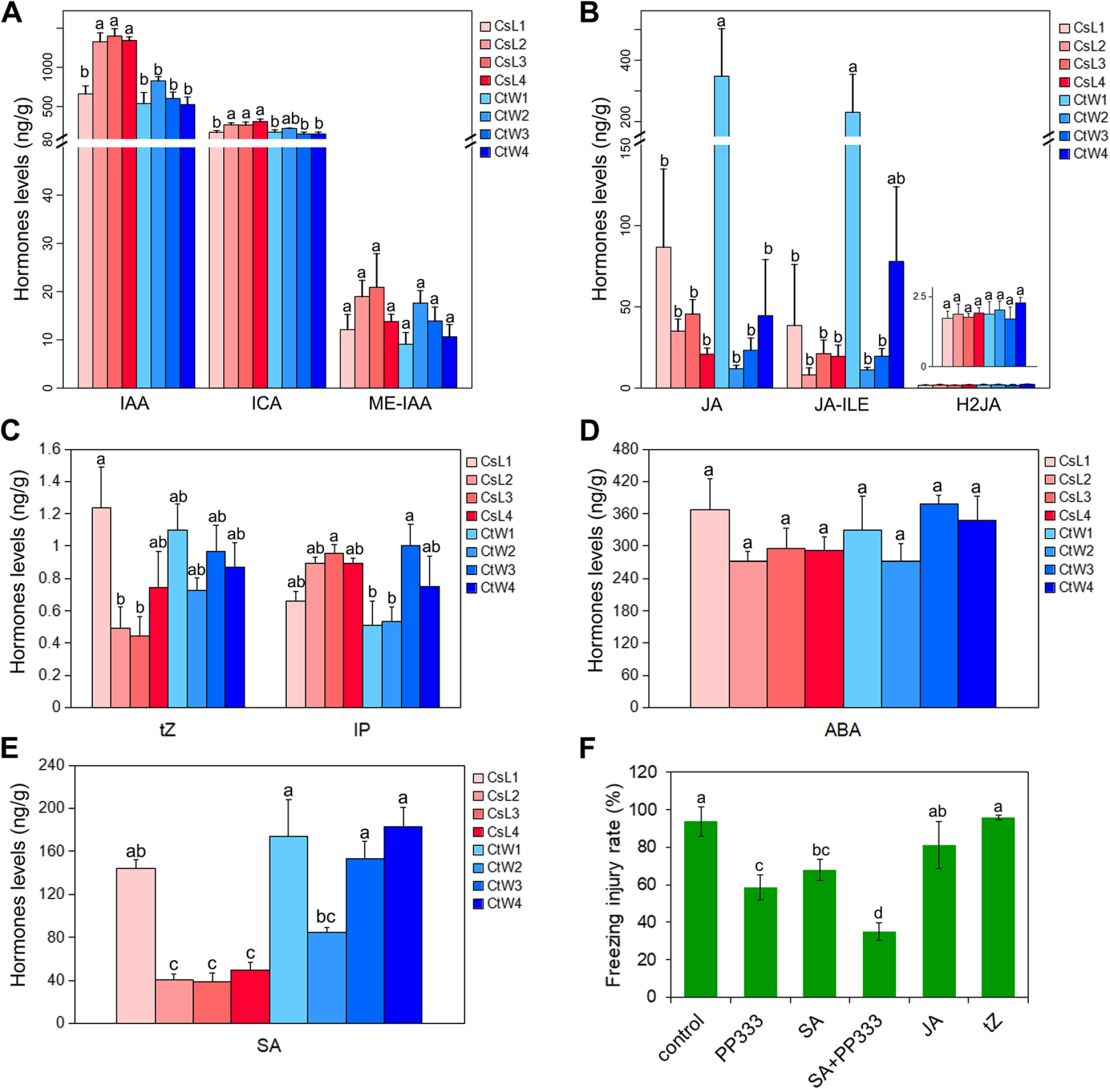
The content and function of hormones in the pistils of apricot kernels under freezing stress. **A**-**E** The hormone content in the pistils of apricot kernels. A Auxin including IAA, ICA and ME-IAA; **B** jasmonic acid including JA, JA-ILE and H2JA; **C** cytokinin including tZ and IP; **D** ABA; **E** SA. **F** The freezing injury rate of the pistils of apricot kernels treated by different hormones under spring frost. Different lowercase letters (a-d) indicate a significant difference (*p* < 0.05). CsL: cold-sensitive ‘Longwangmao’; CtW: cold-tolerant ‘Weixuan 1’. CsL1-4 and CtW1-4 refer to the samples of CsL and CtW treated at different temperatures (20 °C, − 2 °C, − 3 °C and − 4 °C), respectively

To investigate the differences in hormones involved in the response to freezing stress between the pistils of CsL and CtW, the detached flower branches were treated at **−** 2 °C, **−** 3 °C and **−** 4 °C. Thereafter, we determined the content of hormone of the cold**–**untreated (the control) and cold**–**treated pistils for CsL (CsL1, CsL2, CsL3 and CsL4) and CtW (CtW1, CtW2, CtW3 and CtW4) using LC–MS/MS, including auxins, cytokinins (CKs), ABA, JAs and SA. Column chart analysis was performed based on hormone quantification (Fig. 1). The results showed that the production of auxin (IAA and ICA) in the pistils of CsL was significantly induced and higher than that in CtW, and the auxin content in CtW had no obvious response to freezing stress (Fig. 1A). In CtW, the JA and JA-ILE contents were decreased, and those in CsL were not significantly different in response to freezing stress (Fig. 1B). In contrast, the tZ of CKs was reduced in CsL2 and CsL3 and showed no evident change in CtW under freezing stress. Another CK, IP, was only induced by low temperature in CtW3 (Fig. 1C). However, there were no significant differences in the accumulation of JA, JA-ILE, tZ and IP between CsL and CtW under freezing stress (Fig. 1B and C). The content of SA was decreased in CsL and in CtW2 but was not distinctly changed in CtW3 and CtW4 (Fig. 1E). In addition, freezing stress exerted no effect on the content of ME-IAA, H2JA and ABA compared with that seen in the control CsL and CtW (Fig. 1A, B, D).

Meanwhile, the external application of the IAA inhibitor (PP333) and SA significantly reduced the freezing injury rate of the pistils of CsL; this effect was higher than that of single application of the IAA inhibitor or SA. The external application of JA or tZ exerted no effect on the freezing injury rate of the pistils of CsL (Fig. 1F). These results indicated that IAA and SA were involved in the response of the pistils of apricot kernels to freezing stress, and IAA may be one of the factors resulting in the difference in freezing resistance between CsL and CtW.

### Transcriptome analysis of the pistils of freezing stress−treated apricot kernels

To investigate the genetic basis and potential mechanism of the hormone-associated process during freezing stress in the pistils of apricot kernels, RNA-seq data were generated for eight pistil samples (CsL1, CsL2, CsL3, CsL4, CtW1, CtW2, CtW3 and CtW4) under freezing stress and control conditions (Fig. S1; Table S2). A total of 1,139,578,524 clean reads were obtained from all libraries, ranging from 41,637,772 to 60,245,518. The Q30% was more than 91%, and the GC content was on average 46%, indicating that the quality of the RNA-seq data was high. Of the total clean reads, 85.28 to 91.83% were uniquely mapped to the apricot reference genome (Table S3).

**Fig. 2:**
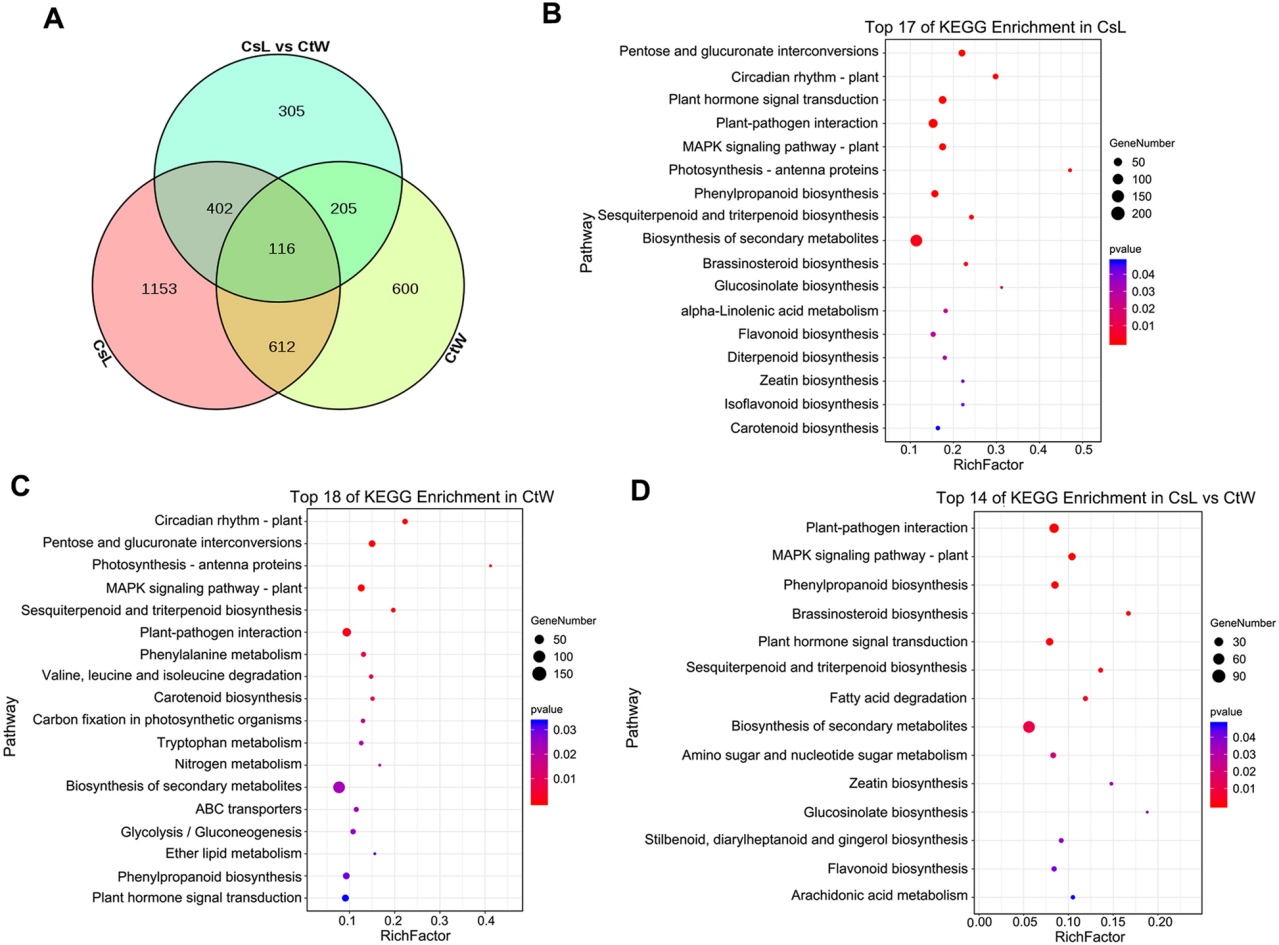
Analysis of DEGs in two apricot kernel cultivars under freezing stress. **A** Venn diagram of DEGs identified in CsL, CtW and CsL vs CtW. **B**-**D** KEGG significantly enriched pathways of DEGs in CsL (**B**), CtW (**C**) and CsL vs CtW (**D**)

The transcriptome profiles of CsL and CtW with and without freezing stress were compared to identify the DEGs. After comparison, a total of 3394 DEGs were obtained, including 2283 DEGs in CsL (CsL1 vs CsL2, CsL1 vs CsL3 and CsL1 vs CsL4), 1533 DEGs in CtW (CtW1 vs CtW2, CtW1 vs CtW3 and CtW1 vs CtW4), and 1028 DEGs in CsL vs CtW (CsL2 vs CtW2, CsL3 vs CtW3 and CsL4 vs CtW4) eliminating inherent differences (CsL1 vs CtW1) (Table 1; Table S4). There were 728 shared DEGs in two group comparisons (CsL and CtW), which were 47.5% of the total DEGs in CtW and 31.9% of the total DEGs in CsL (Fig. 2A). Of these shared DEGs, only 15.9% (116) were also present in CsL vs CtW, indicating that these genes were not only regulated in both CsL and CtW in response to freezing stress, but are also regulated differently in CsL and CtW. There were more DEGs in CsL than in CtW at − 2 °C and − 3 °C. In the two-group comparisons of CsL and CsL vs CtW, the highest number of DEGs was CsL1 vs CsL3 and CsL3 vs CtW3, while the highest in CtW was CtW1 vs CtW2. The DEGs with upregulated expression were more abundant than those with downregulated expression in CsL and CtW treated at different low temperatures, whereas the downregulated DEGs were more abundant than the upregulated DEGs in CsL vs CtW group under − 2 °C and − 3 °C conditions (Table 1). Moreover, the upregulated DEGs in CsL were more abundant than those in CtW.

**Table 1:** Differentially expressed genes (DEGs) in ‘Longwangmao’ and ‘Weixuan 1’ under freezing stress

Groups	Total DEGs		Downregulated DEGs	Upregulated DEGs
CsL1 vs CsL2	2283	1168	337	831
CsL1 vs CsL3		1438	251	1187
CsL1 vs CsL4		729	105	624
CtW1 vs CtW2	1533	876	347	529
CtW1 vs CtW3		638	282	356
CtW1 vs CtW4		676	244	432
CsL2 vs CtW2	1028	208	156	52
CsL3 vs CtW3		678	465	213
CsL4 vs CtW4		269	98	171
CsL1 vs CtW1		72	45	27

### Functional analysis of DEGs of the pistils of freezing stress–treated apricot kernels

To identify the biological functions of DEGs in CsL and CtW under freezing stress, KEGG enrichment analysis was conducted. In CsL, CtW and CsL vs CtW, the MAPK signaling pathway-plant, plant hormone signal transduction, biosynthesis of secondary metabolites, plant–pathogen interaction and phenylpropanoid biosynthesis, which may play an important role in response to freezing stress, were all enriched (*P* < 0.05); the number of DEGs enriched in the biosynthesis of secondary metabolites were the highest (Fig. 2B-D). Additionally, BR biosynthesis, zeatin biosynthesis and flavonoid biosynthesis were enriched in CsL and CsL vs CtW. The pathways of photosynthesis, circadian rhythm and carotenoid biosynthesis were both enriched in CsL and CtW. Furthermore, phenylalanine metabolism, nitrogen metabolism and ABC transporters were also enriched in CtW (Fig. 2B-D). The results indicated that freezing stress could regulate the complex biological pathways of apricot kernels, and there were the shared and different pathways between CsL and CtW that may function in response to freezing stress.

The plant hormone signal transduction may contribute to differences in frost resistance of CsL and CtW. There were 73, 38 and 33 DEGs in plant hormone signal transduction pathways in CsL, CtW and CsL vs CtW, respectively (Fig. S2). A total of 26 DEGs were present in both CsL and CtW, suggesting that they may be involved in the response of apricot kernels to freezing stress. Six of these DEGs were present in all three groups (CsL, CtW and CsL vs CtW), and these genes may result in the different freezing resistance between CsL and CtW (Fig. S2). Furthermore, the number of DEGs in auxin or SA signal transduction pathways were the highest in all three groups (Table S5).

### Identification of co-expression network and hub genes related to hormones

**Fig. 3:**
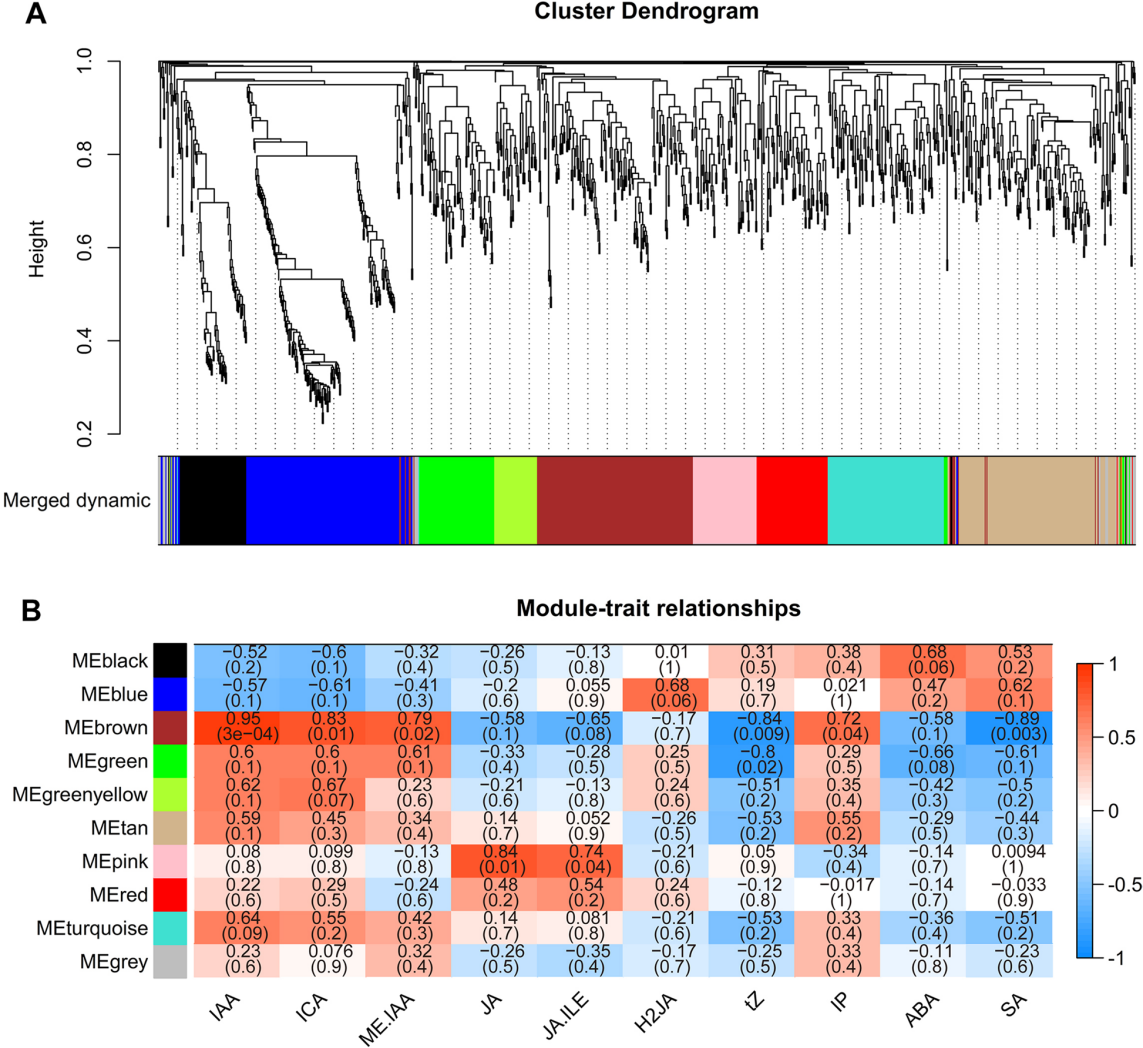
DEGs and hormone correlation analysis in two apricot kernel cultivars. **A** Cluster dendrogram showing 10 gene co-expression modules labeled with different colors by WGCNA. **B** The heatmap of module-hormone relationships. Each row represents a module indicated by different colors. The color key from blue to red represents correlation values from − 1 to 1

To detect the differential co-expressed genes associated with hormones between CsL and CtW under freezing stress, DEG expression profiles (1028) were examined using WGCNA. Ten distinct modules assigned with different colors were identified based on the co-expression patterns of genes (Fig. 3A). Of the 10 co-expressed gene modules, the brown module containing 179 genes showed a significant positive association with IAA (*r* = 0.95, *P* = 3e-04) and ICA (*r* = 0.83, *P* = 0.01), and a significant negative association with tZ (*r* = − 0.84, *P* = 0.009) and SA (*r* = − 0.89, *P* = 0.003). The pink module containing 68 genes was positively correlated with JA (*r* = 0.84, *P* = 0.01) (Fig. 3B).

**Fig. 4:**
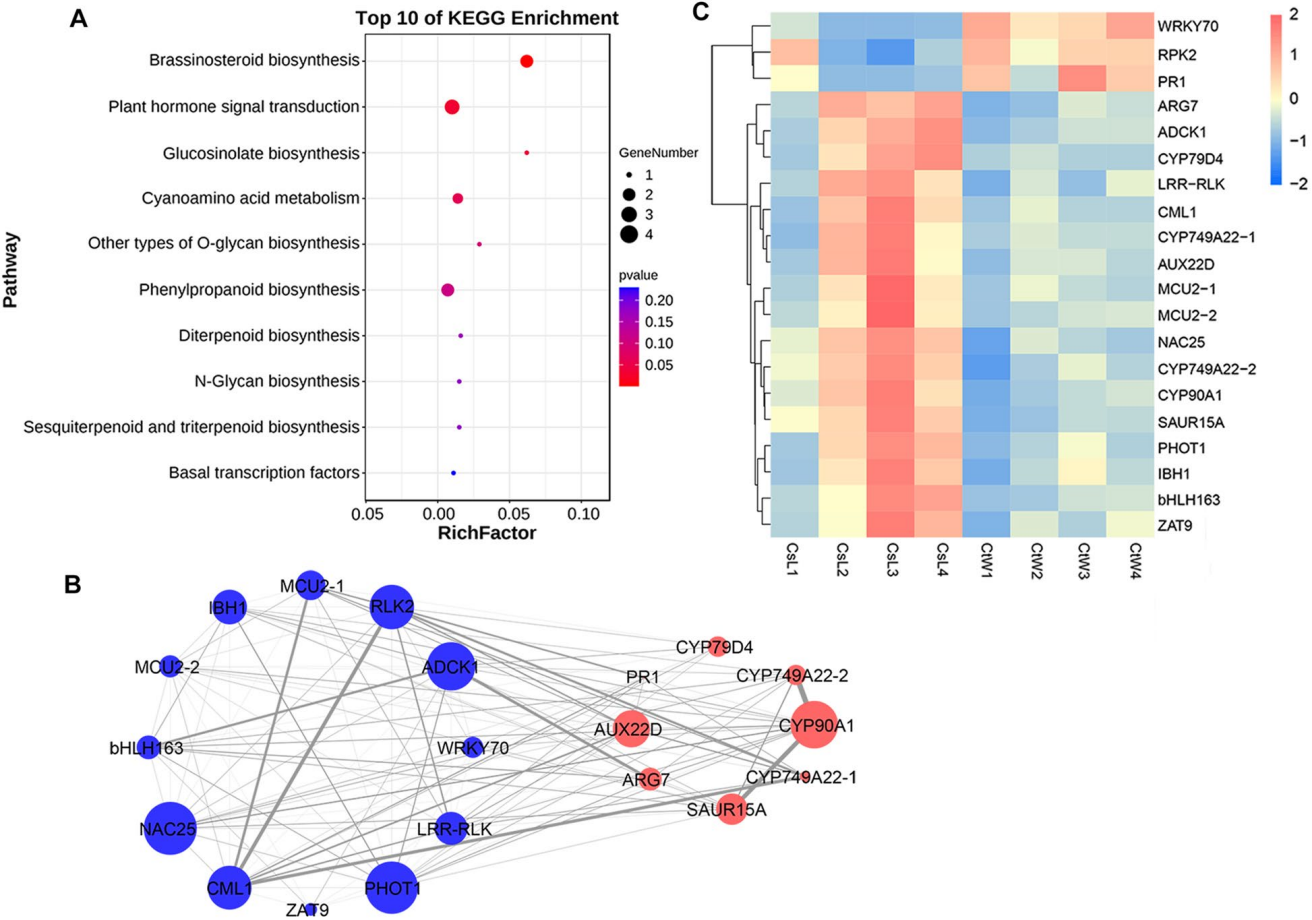
Enrichment analysis and co-expression network of hub genes in the brown module associated with IAA. **A** The KEGG enrichment pathway of hub genes identified in the brown module. **B** Co-expression network of selected hub genes. Blue nodes represent transcription factors, protein kinases and Ca^2+^ related proteins; red nodes represent genes in significantly enriched pathways of hub genes. The size of nodes is based on kME. The width of edges is based on weight. **C** The expression heatmap of hub genes in the co-expression network

To identify the candidate genes involved in the auxin pathway that evidently differed between CsL and CtW, 65 hub genes (kME > 0.9 and GS > 0.2) in the brown module were identified, which had high intramodular connectivity (Table S6). The KEGG enrichment analysis revealed that the hub genes were significantly enriched in the BR biosynthesis, plant hormone signal transduction and glucosinolate biosynthesis (Fig. 4A). Combined with the annotation information, 20 hub genes were used to construct the co-expressed network, including 5 TFs (NAC25, WRKY70, IBH1, ZAT9 and bHLH163), four protein kinases (ADCK1, LRR-RLK, RLK2 and PHOT1), and three Ca^2+^ related genes (CML1, MCU2-1 and MCU2-2) and genes in significantly enriched pathways (auxin signaling pathway: ARG7, SAUR15A and AUX22D; BR biosynthesis: CYP749A22-1, CYP749A22-2 and CYP90A1; glucosinolate biosynthesis: CYP79D4) (Fig. 4B). One genes (CYP90A1) involved in the BR biosynthesis, one TF (NAC25) and two protein kinases (ADCK1 and PHOT1) showed higher connectivity within the co-expressed network, which may play critical roles in auxin-associated processes resulting in different frost resistance of CsL and CtW.

In the co-expression network, there were many interconnections among protein kinases-hormone related genes (HRGs), protein kinases-TFs and Ca^2+^ related genes-HRGs. Most protein kinases were positively correlated with other genes, such as LRR-RLK-CML1, ADCK1-ARG7, ADCK1-bHLH163 and PHOT1-IBH1, whereas RLK2 was negatively related to CML1, AUX22D and CYP749A22-1. The hub genes in the auxin signaling pathway were positively correlated with most other genes, such as AUX22D-CML1, ARG7-bHLH163, SAUR15A-CYP90A1, and SAUR15A-CYP749A22-2 (Fig. 4B and C). These findings implied that there may be some regulatory relationships between these genes that affect the freezing resistance of apricot kernels.

### Genes related to auxin synthesis and signaling pathway

**Fig. 5:**
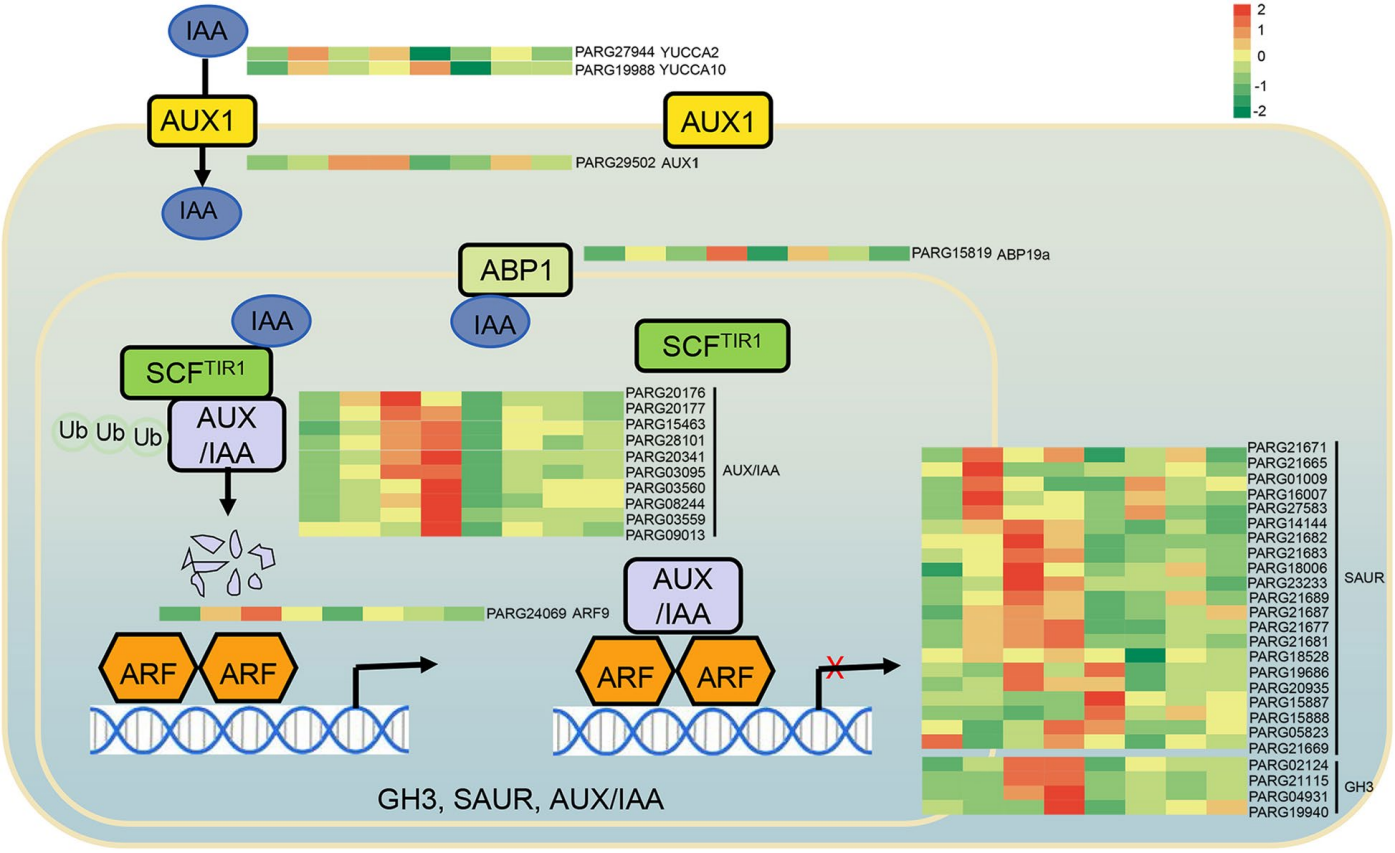
DEGs in the auxin signaling pathway in response to freezing stress. The grids (from left to right) refer to the expression level of DEGs in CsL and CtW under different temperature (20 °C, − 2 °C, − 3 °C and − 4 °C) conditions

Genes involved in ausin synthesis and signal transduction pathways may exert considerable impact on the freezing resistance of apricot kernels. Two indole-3-pyruvate monooxygenases (YUCCA) (PARG27944 and PARG19988), showed differential expression patterns in CtW treated with different low temperatures and CsL vs CtW (Fig. 5; Table S4). Thirty-eight DEGs related to the auxin signal transduction pathway were identified. The expression of one auxin transporter protein AUX1 (PARG29502) and one auxin-binding protein ABP19a (PARG15819) were upregulated in CsL under freezing stress. The expression of 10 *AUX/IAA* genes and one auxin response factor (*PARG24069*, *ARF9*) were induced by freezing stress. Nine (except *PARG09013*), four (*PARG03560*, *PARG15463*, *PARG20341* and *PARG28101*) and five (*PARG09013*, *PARG15463*, *PARG20176*, *PARG20341* and *PARG28101*) *AUX/IAA* genes were differentially expressed in CsL, CtW and CsL vs CtW, respectively. Meanwhile these *AUX/IAA* genes and *ARF9* had higher expression levels in CsL than those in CtW. Twenty-one *SAUR* genes were differentially expressed under different low-temperature conditions in the pistils of apricot kernels. Twelve *SAUR* genes were induced in CsL, of which four (*PARG21671*, *PARG01009*, *PARG16007* and *PARG27583*) had high expression levels in CsL2, five (*PARG21683*, *PARG18006*, *PARG23233*, *PARG19686* and *PARG20935*) had high expression levels in CsL3, and one (*PARG21681*) had high expression level in CsL4. The expression of one *SAUR* gene (*PARG21669*) was downregulated by freezing stress in CsL. In CtW, two *SAUR* genes (*PARG19686* and *PARG15887*) were downregulated, and three (*PARG21671*, *PARG21687* and *PARG21689*) were upregulated by freezing stress (Fig. 5). In addition, seven *SAUR* genes were differently expressed between CsL and CtW, including *PARG19686*, whose expression was up-regulated in CsL and down-regulated in CtW (Fig. 5; Table S5). Of four indole-3-acetic acid-amido synthetases GH3, three (*PARG02124*, *PARG21115* and *PARG04931*) exhibited increasing trends in response to freezing stress, especially at − 3 °C and − 4 °C in CsL. However, these *GH3* genes showed no significant expression changes in CtW. One *AUX/IAA* gene (*PARG20176*, *AUX22D*) and two *SAUR* gene (*PARG21682*, *SAUR15A; PARG21677*, *ARG7*) were also present in the brown module and were strongly correlated (r = 0.95) with IAA content. Meanwhile, the expression patterns of most genes were consistent with that of the IAA content in CsL and CtW in response to freezing stress, suggesting that IAA may play a negative role in regulating the freezing resistance of CsL.

### qRT-PCR validation of DEGs

**Fig. 6:**
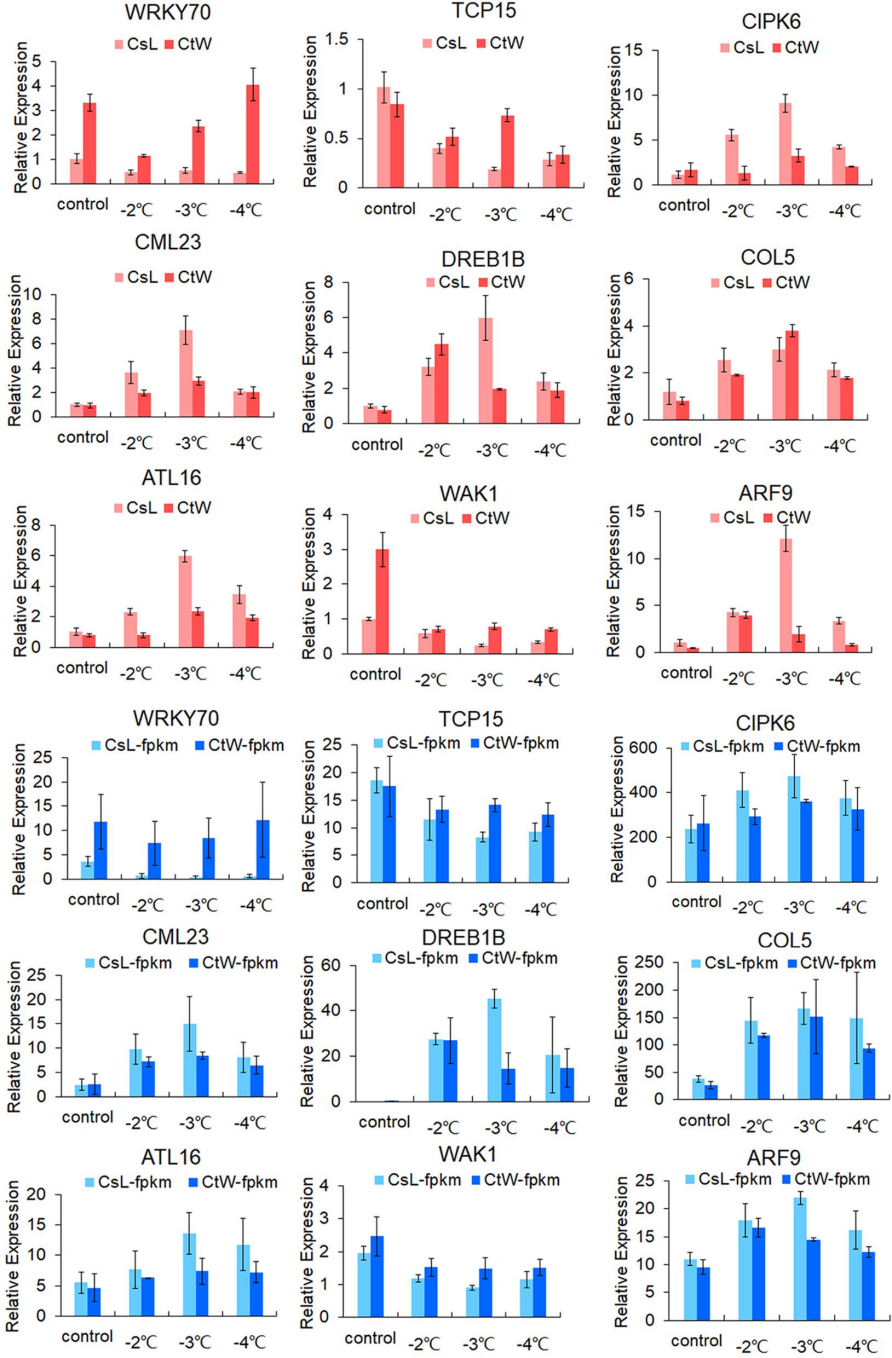
qRT-PCR analysis of nine hub genes in the gene network. *18S* was used as the internal control. Error bars indicate SD

For the validation of the gene expression pattern of DEGs in two apricot kernel cultivars, nine genes were selected for qRT-PCR assays (Fig. 6). The expression of *ARF9*, *COL5*, *DREB1B*, *CML23*, *CIPK6* and *ATL16* were induced in CsL and CtW under freezing stress; however those of *CIPK6* and *ATL16* showed no evident change in CtW2. In addition, *ARF9*, *CML23*, *CIPK6* and *ATL16* had higher expression levels in CsL under freezing stress. The expression levels of *WRKY70*, *TCP15* and *WAK1* decreased in CsL under freezing stress. The expression profiles of these genes in qRT-PCR were consistent with those in the RNA-seq results, verifying the credibility of the RNA-seq data.

## Discussion

Freezing temperature is an important environmental factor that impacts the metabolism, growth and stress responses of plants and is regulated by various messenger molecules and gene regulators. Plant hormone signaling is intricately linked to molecular and physiological responses to low temperature. In this study, using WGCNA integrating transcriptome and hormone data for the first time, the complex genetic factors governing hormone pathways underlying the variation in freezing resistance of apricot kernel were identified.

### Hormone signaling and regulation under freezing stress

Our study showed that freezing stress increased the auxin (IAA and ICA) content in CsL but decreased the JA content (JA and JA-ILE) in CtW and the cytokinin tZ and SA contents in CsL (Fig. 1). With a similar trend as auxin, auxin biosynthetic genes (*YUCCA2* and *YUCCA10*) were induced in CsL in response to freezing stress (Fig. 5). It has been reported that in *Arabidopsis thaliana,* the auxin level was decreased in response to cooler temperature via repression of biosynthetic gene expression, but in rice cold stress increased the auxin level [30, 31]. Under low temperature, the change trends of tZ and SA in the pistils of CsL are opposite to those in Arabidopsis*,* whereas the change trend of JA was consistent with that in maize and deciduous trees and opposite to that in tea plants (Fig. 1) [16]*.* These results suggest that hormone responses to cold stress are species specific or are different at different treatment stages. Based on the comparison of hormone content in two apricot kernel cultivars and the experiment of external hormone application, IAA was found to potentially play a vital role in the different freezing resistances of CtW and CsL.

Hormone signaling and its important components are involved in regulating plant cold tolerance, such as BR-BZR1/BES1, auxin-AUX/IAA and ABA-protein kinase SnRK2/OST1 [16, 19, 32]. Here, the pathway of plant hormone signal transduction was significantly enriched in DEGs across the three comparison groups of apricot kernel pistils (Fig. 2B-D), indicating that the plant hormone signaling pathway not only played an important role in regulating the freezing resistance of apricot kernel but was also one of the factors resulting in the different freezing resistances between CtW and CsL. Previous studies have shown that auxin is involved in the cold stress response, and AUX/IAA genes function as hubs that integrate stress pathways and the auxin regulatory network [17, 33, 34]. In this study, three auxin signaling pathway genes (*SAUR15A*, *AUX22D* and *ARG7*) were found to be highly correlated with IAA content under freezing stress (Fig. 4), suggesting that auxin may be involved in freezing stress responses via these genes and result in the different freezing resistance of CsL and CtW. In rice, genome-wide analysis of auxin-responsive genes revealed that the upregulation and downregulation of the expression of *GH3*, *AUX/IAA*, *SAUR* and *ARF* genes were present under cold stress [35]. Consistent with this, the *SAUR* (especially *PARG19686*) and *GH3* genes (such as *PARG21115* and *PARG04931*), were differently regulated by freezing stress in two apricot kernel cultivars (Fig. 5). Thus, we believe that auxin signaling and its important components play a key role in the response to freezing stress in the pistils of apricot kernel to balance freezing stress tolerance and growth [36].

In addition, several BR biosynthetic genes such as *CYP90A1* were highly associated with IAA content (Fig. 4). BR signaling, which can be regulated by cold stress, usually functions in seed germination by integrating GA and ABA signaling [16, 37]. Here, the co-expression relationships of SAUR15A-CYP90A1 and SAUR15A-CYP749A22-2 were found, implying that the crosstalk of BR and auxin under freezing stress.

### Regulators and network involved in the hormone pathway under freezing stress

Numerous genes are involved in the cold response and are often regulated via coordinated expression, and hence, gene networks can be analyzed based on correlation-based models. We identified one gene module/network that was highly correlated with auxin, and the hub genes within this module included TFs, protein kinases, Ca^2+^ related genes and HRGs, which may function in regulating freezing resistance (Fig. 4B; Table S6).

Ca^2+^, a ubiquitous secondary messenger, plays a key role in the cold response of plants, and several Ca^2+^ transporters, channels and sensors were shown to be involved in cold-evoked signal transduction [38, 39]. Consistent with previous studies, Ca^2+^ uniporter proteins (MCU2-1 and MCU2-2) and sensor proein CML1 were identified as hub genes in the co-expression network under freezing stress and had correlations with AUX22D, LRR-RLK and RLK2 (Fig. 4). Several RLKs have been reported to function in regulating plant tolerance to cold stress. In Arabidopsis, CRLK1 (Ca^2+^/calmodulin-regulated receptor-like kinase) and CRLK2 positively regulate freezing tolerance via MAPK pathway, and possibly couple with a cold-response RLK to perceive the cold signal [13]. Thus, Ca^2+^ uniporter proteins and sensors may work together with RLKs to sensor or influence Ca^2+^ influx to trigger cold signals. In addition, the cold signal is also regulated by light, and blue light receptor PHOT1 can affect blue light-stimulated changes in cytosolic Ca^2+^ in Arabidopsis [40]. Meanwhile, PHOT1 was found to be highly related to IAA content and co-expressed with *ARG7* (Fig. 4). Notably, there was a link between protein kinases, light and Ca^2+^ signaling to regulate auxin-mediated cold response of the pistils of apricot kernels.

Transcriptional regulation plays a major role in response to low temperatures, and various TFs such as NAC and WRKY have been identified to modulate the cold tolerance of plants via CBF-dependent and CBF-independent pathways [41]. The expression of *WRKY70*, acting as a hub gene in co-exspression network, was downregulated in CsL, in contrast to that of other hub TFs (Fig. 4), suggesting that the function of WRKY70 in regulating the freezing resistance of apricot kernel pistils may also be opposite with other hub TFs. Meanwhile, the expression pattern correlations of TF-protein kinases and TF-HRGs such as PHOT1-IBH1 and ARG7-bHLH163 were identified in this network, indicating that auxin may participate in the freezing response via complex gene networks in apricot kernels.

## Conclusions

We used transcriptome and hormone profiles to investigate hub genes and gene co-expression network regulating the freezing resistance of apricot kernel pistils. IAA and SA were identified to function in freezing resistance of apricot kernel pistils, and IAA might result in different freezing resistances between two apricot cultivars. Their correlative hub gene network was determined, and within this network, three genes in the auxin signaling pathway and three genes in the BR biosynthesis pathway were identified. Some hub genes were newly identified to be involved in response to freezing stress. Our study provides new ideas for frost prevention measures in apricot varieties.

## Supplementary Information

**Additional file 1: Fig. S1.** Clustering and PCA of transcriptomes of CsL and CtW. **Fig. S2.** Venn diagram of the DEGs related to plant hormone signaling transduction identified in CsL, CtW and CsL vs CtW. **Table S1.** List of primers used for qRT-PCR. **Table S3**. Summary of mapping transcriptome reads to reference sequence.

**Additional file 2: Table S2.** The expression information of genes in the transcriptome of two apricot kernel cultivars.

**Additional file 3: Table S4.** The raw expression information of DEGs in two apricot kernel cultivars under freezing stress.

**Additional file 4: Table S5.** The expression level of DEGs related to plant hormone signaling transduction in two apricot kernel cultivars under freezing stress.

**Additional file 5: Table S6.** Annotation of hub genes in the brown module.

## Data Availability

The data supporting the results are concluded in the article and supplementary information files. The RNA-seq data were deposited in NCBI SRA with the accession number of PRJNA854214.
