# Supplementary material for: Identification of key gene networks related to the freezing resistance of apricot kernel pistils by integrating hormone phenotypes and transcriptome profiles
Source: BMC Plant Biol. 2022 Nov 15;22:531. doi: 10.1186/s12870-022-03910-4 (PMC9664786; doi:10.1186/s12870-022-03910-4)
Supplement: Supplementary file 1 — Additional file 1: Fig. S1. Clustering and PCA of transcriptomes of CsL and CtW. Fig. S2. Venn diagram of the DEGs related to plant hormone signaling transduction identified in CsL, CtW and CsL vs CtW. Table S1. List of primers used for qRT-PCR. Table S3. Summary of mapping transcriptome reads to reference sequence. [file 12870_2022_3910_MOESM1_ESM.doc]

**Identification of key gene networks related to the freezing resistance of apricot kernel pistils by integrating hormone phenotypes and transcriptome profiles**

Xiaojuan Liu, Huihui Xu, Dan Yu, Quanxin Bi, Haiyan Yu, Libing Wang*

State Key Laboratory of Tree Genetics and Breeding, Research Institute of Forestry, Chinese Academy of Forestry, 100091 Beijing, China.

*: Correspondence: [wlibing@caf.ac.cn](mailto:wlibing@caf.ac.cn)


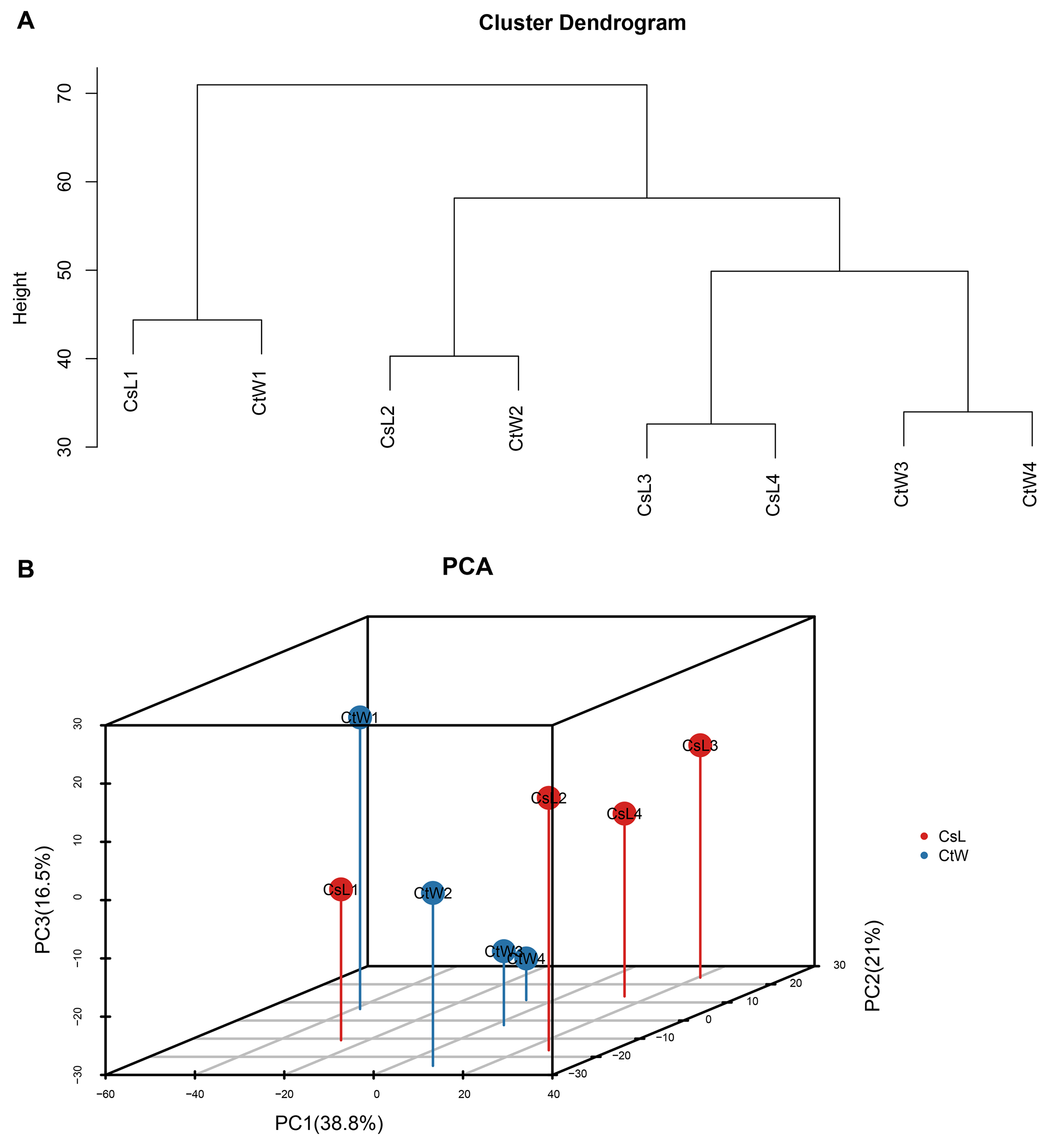


**Fig. S1.** Clustering and PCA of transcriptomes of CsL and CtW. (A) The cluster dendrogram of CsL and CtW treated with different temperatures. (B) The PCA analysis of CsL and CtW treated with different temperatures. CsL: cold-sensitive ‘Longwangmao’; CtW: cold-tolerant ‘Weixuan 1’. CsL1-4 and CtW1-4 refer to the samples of CsL and CtW treated at different temperatures (20 °C, −2 °C, −3 °C and −4 °C), respectively.


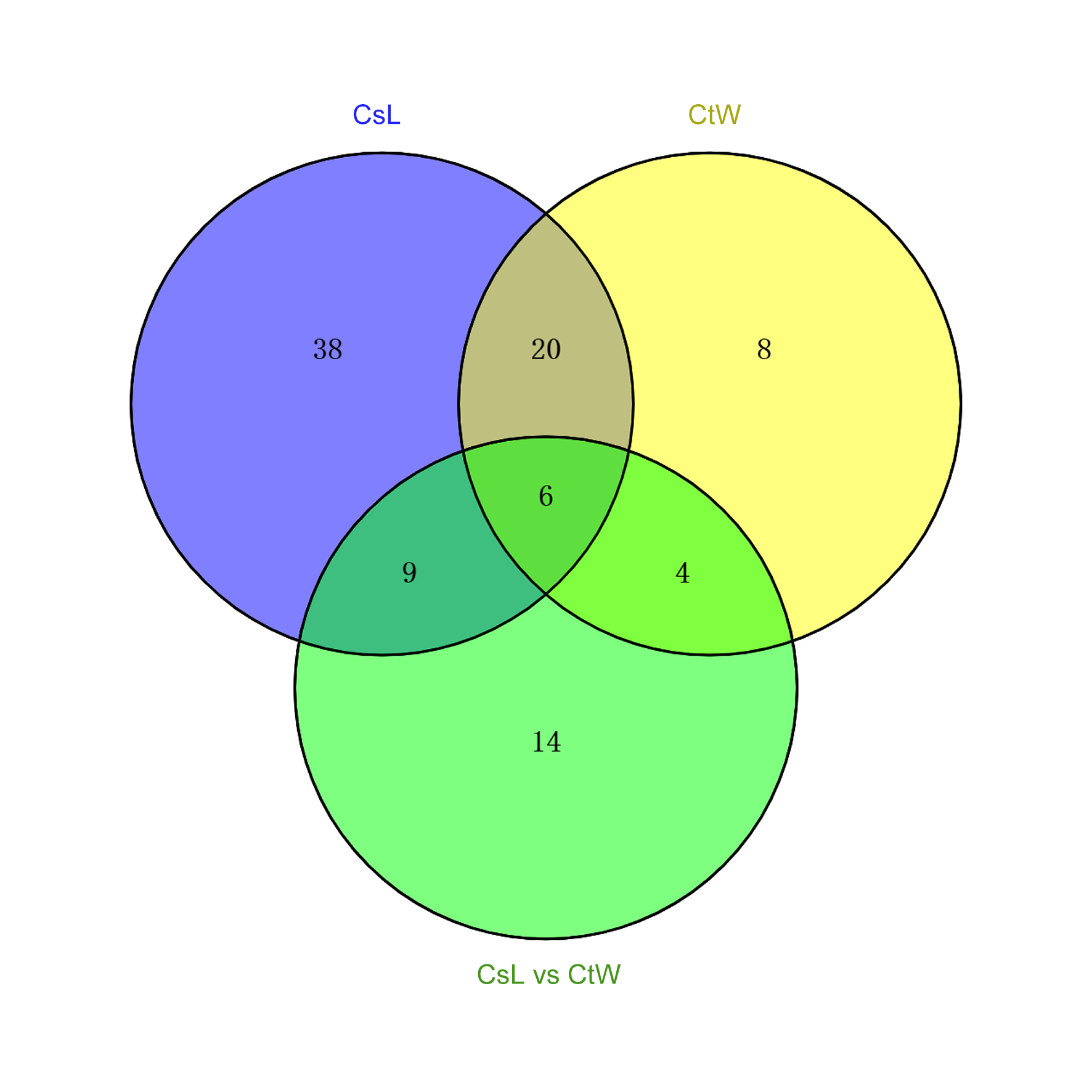


**Fig. S2.** Venn diagram of the DEGs related to plant hormone signaling transduction identified in CsL, CtW and CsL vs CtW.

**Table S1. List of primers used for qRT-PCR**

| *ARF9* (*PARG24069*) | F: CACCACCGAGTGTTGCTACTGAAG |
| --- | --- |
|  | R: ACCACCTGTCTGAGATAGCCATCC |
| *COL5* (*PARG24308*) | F: ATCGGGAGGCTAGGGTTTTGAGG |
|  | R: GATTTCGGTTTCGGTGCGTTTCG |
| *WRKY70* (*PARG29164*) | F: AGGAGATGTTGAGTCGGCAGAGG |
|  | R: ACCCACCACCACTACTATTCCCTTG |
| *DREB1B* (*PARG23730*) | F: CAAGTGGGTGTGTGAGTTGAGAGAG |
|  | R: CTTCCCTCTGAACGCCAATGCC |
| *TCP15* (*PARG24815*) | F: GTGGAGGTAACGACGGCGATAAC |
|  | R: ACTTGCTTGCTGGGACTCTGAAAC |
| *CML23* (*PARG14409*) | F: CATAATGGCGGAGTTCGACAGAGAC |
|  | R: GCCGAGATCAGTCCGTTCTTGTC |
| *CIPK6* (*PARG28297*) | F: GGGTCACGGTACTTTCGCCAAG |
|  | R: ACCTTCTCTTTGCCCACCACTTTC |
| *WAK1* (*PARG27690*) | F: AGGTCAAACTATGATGGCTGCGATG |
|  | R: GAATGTGAGTGGTGACGGAAGTGAG |
| *ATL16* (*PARG14104*) | F: GTGTCAGCAAGCTCCGATCCAAG |
|  | R: TGGTGATGGTGGTAGAAGAGTAGGG |
| *18S* | F: ACACGGGGAGGTAGTGACAA |
|  | R: CCTCCAATGGATCCTCGTTA |

**Table S2.** Summary of mapping transcriptome reads to reference sequence.

| Variety | Treatment | Sample | Clean reads | GC (%) | Q30 (%) | Unique mapped (%) |
| --- | --- | --- | --- | --- | --- | --- |
| ‘Longwangmao’ (CsL) | CsL1 | CsL11 | 60,245,518 | 45.92 | 92.15 | 91.47 |
|  |  | CsL12 | 48,251,936 | 45.93 | 91.73 | 90.81 |
|  |  | CsL13 | 48,950,240 | 46.25 | 91.86 | 91.31 |
|  | CsL2 | CsL21 | 50,529,190 | 45.85 | 91.66 | 90.79 |
|  |  | CsL22 | 53,214,736 | 45.8 | 91.99 | 90.86 |
|  |  | CsL23 | 49,869,406 | 45.81 | 92.89 | 91.10 |
|  | CsL3 | CsL31 | 45,975,804 | 45.89 | 91.99 | 90.61 |
|  |  | CsL32 | 45,188,742 | 45.82 | 92.08 | 90.50 |
|  |  | CsL33 | 42,924,054 | 45.9 | 91.94 | 89.56 |
|  | CsL4 | CsL41 | 42,680,492 | 46.46 | 91.86 | 85.28 |
|  |  | CsL42 | 48,642,822 | 45.68 | 92.32 | 89.40 |
|  |  | CsL43 | 47,419,520 | 45.83 | 92.35 | 89.05 |
| ‘Weixuan 1’  (CtW) | CtW1 | CtW11 | 41,850108 | 45.93 | 92.29 | 89.62 |
|  |  | CtW12 | 41,637,772 | 46.20 | 92.34 | 91.59 |
|  |  | CtW13 | 50,142,906 | 46.10 | 92.29 | 91.23 |
|  | CtW2 | CtW21 | 45,560,166 | 46.15 | 92.18 | 91.07 |
|  |  | CtW22 | 47,698,510 | 45.80 | 91.70 | 90.31 |
|  |  | CtW23 | 44,930,806 | 46.07 | 92.93 | 91.83 |
|  | CtW3 | CtW31 | 45,458,176 | 45.97 | 92.26 | 91.18 |
|  |  | CtW32 | 44,438,086 | 46.16 | 92.35 | 91.04 |
|  |  | CtW33 | 45,035,024 | 46.43 | 92.43 | 90.53 |
|  | CtW4 | CtW41 | 50,778,572 | 45.84 | 92.77 | 91.12 |
|  |  | CtW42 | 52,775,692 | 45.76 | 92.05 | 91.40 |
|  |  | CtW43 | 45,380,246 | 45.96 | 91.95 | 90.73 |
